# Occurrences of Workplace Violence Related to the COVID-19 Pandemic, United States, March 2020 to August 2021

**DOI:** 10.3390/ijerph192114387

**Published:** 2022-11-03

**Authors:** Suzanne M. Marsh, Carissa M. Rocheleau, Eric G. Carbone, Daniel Hartley, Audrey A. Reichard, Hope M. Tiesman

**Affiliations:** 1 Surveillance and Field Investigations Branch, Division of Safety Research, National Institute for Occupational Safety and Health, Morgantown, WV 26505, USA; 2 Field Research Branch, Division of Field Studies and Engineering, National Institute for Occupational Safety and Health, Cincinnati, OH 45226, USA; 3 Office of Extramural Programs, Office of the Director, National Institute for Occupational Safety and Health, Morgantown, WV 26505, USA; 4 Analysis and Field Evaluations Branch, Division of Safety Research, National Institute for Occupational Safety and Health, Morgantown, WV 26505, USA

**Keywords:** COVID-19, SARS-CoV-2, workplace violence, media scraping, employee safety, assault, mask policy, news media

## Abstract

As businesses dealt with an increasingly anxious public during the COVID-19 pandemic and were frequently tasked with enforcing various COVID-19 prevention policies such as mask mandates, workplace violence and harassment (WPV) emerged as an increasing important issue affecting worker safety and health. Publicly available media reports were searched for WPV events related to the COVID-19 pandemic that occurred during 1 March 2020, and 31 August 2021, using Google News aggregator services scans with data abstraction and verification. The search found 408 unique WPV events related to COVID-19. Almost two-thirds involved mask disputes. Over half (57%) of the 408 events occurred in retail (38%) and food service (19%). We also conducted a comparison of events identified in this search to a similar study of media reports between March 2020 to October 2020 that used multiple search engines to identify WPV events. Despite similar conclusions, a one-to-one comparison of relevant data from these studies found only modest overlap in the incidents identified, suggesting the need to make improvements to future efforts to extract data from media reports. Prevention resources such as training and education for workers may help industries de-escalate or prevent similar WPV events in the future.

## 1. Introduction

Since the beginning of the Coronavirus Disease-2019 (COVID-19) pandemic caused by severe acute respiratory syndrome coronavirus 2 (SARS-CoV-2), many statewide and/or business-wide mandates were implemented including: masks and vaccination requirements; distancing protocols; and household goods purchasing limits to address supply shortages. For example, an average of 53% of U.S. states had a mask mandate in place for either a partial or full month from April 2020 to August 2021 [1]. Averages ranged from a high of 78% of states with mandates in January of 2021 to a low of 14% of states with mandates in August 2021. As these mandates were being implemented, workers in critical infrastructure industries such as health care, food service, retail, and public transportation were deemed essential and were tasked with enforcing various COVID-19 prevention policies such as mask mandates. As some customers, clients, and patients became more anxious about COVID-19 and more frustrated with the COVID-19 prevention policies that were being implemented, workplace violence and harassment (WPV) emerged as an important issue affecting worker safety and health [2,3].

Over the last 100 years, there have been other instances of WPV occurring during epidemics and pandemics. From the 1830s through the 1910s, those involved in infection control and other public health activities related to Cholera faced mistrust and hostility [4]. The 1918–1920 H1N1 influenza pandemic, which also involved wide-scale mask mandates, resulted in organized as well as episodic opposition and violence against those enforcing these mandates [5]. Finally, the 2014–2016 Ebola virus outbreak resulted in physical attacks and threats to healthcare workers, journalists, government officials, and others assisting those who were sick [4]. It is not surprising then that history repeated itself during the current pandemic. A study of the current pandemic conducted by One Fair Wage [6] found that over three quarters of the 1675 workers surveyed reported experiencing or witnessing violence from customers as COVID-19 prevention policies and safety protocols were enforced.

WPV involves violent acts, such as physical assaults and threats of assault, directed toward persons at work or on duty [7,8]. Unlike physical assaults (i.e., hitting, slapping, kicking, pushing, choking, grabbing, spitting on or at someone, or other physical contact with the intent of causing injury or harm), the intent of threats and verbal assaults is not necessarily to cause physical harm, but to cause negative emotions in the person being assaulted. The National Institute for Occupational Safety and Health (NIOSH) has been engaged in WPV research since the 1980s [7,9,10,11]. However, media reports alerted NIOSH to emerging WPV issues unique to the enforcement of COVID-19 prevention policies. Because there was a dearth of literature on WPV related to COVID-19 prevention policies in the U.S. in 2020, NIOSH undertook two projects to capture WPV events to provide a more thorough understanding of the circumstances surrounding WPV related to the current COVID-19 pandemic. The first of these by Tiesman et al. [12], described elsewhere [12], used two search strategies and five search engines (i.e., news.Google.com, news.yahoo.com, bing.com/news, duckduckgo.com, and Buzzsumo.com) to retrospectively identify news events occurring from March 2020 to October 2020, and found that mask mandates were associated with a large proportion of violent incidents early in the pandemic.

The current project was initiated within the NIOSH Emergency Preparedness and Response Office, Disaster Science Responder Research Program (DSRR). Early in the pandemic, the DSRR identified a group of critical topic areas associated with SARS-CoV-2 and COVID-19 including WPV, how workers are infected, whether vaccinated workers are protected against re-infection, how to decrease transmission, effectiveness of physical barriers, and many others. While most of these areas could be addressed through literature reviews, the DSRR determined that it was necessary to obtain information about WPV events through a rapid case identification approach using media stories identified from Google News aggregator service. Based on biweekly news searches, this near real-time case identification approach was used to identify WPV events related to the COVID-19 pandemic that occurred during 1 March 2020, and 31 August 2021. This manuscript presents an analysis of these media reports on WPV to describe trends, the context of each event, and characteristics of the perpetrators and victims. We also obtained event descriptions from the prior media analysis of WPV related to COVID-19 [12] and compared events of WPV identified in each study to evaluate inter-method reliability.

Our aims in this study were to (1) describe the aggregated media reports of WPV related to COVID-19 that were collected from a near real-time review methodology that was used to help inform Centers for Disease Control and Prevention (CDC) COVID-19 response internally; and (2) compare the events identified from both case identification projects for the period where the studies overlapped.

## 2. Materials and Methods

**Figure 1:**
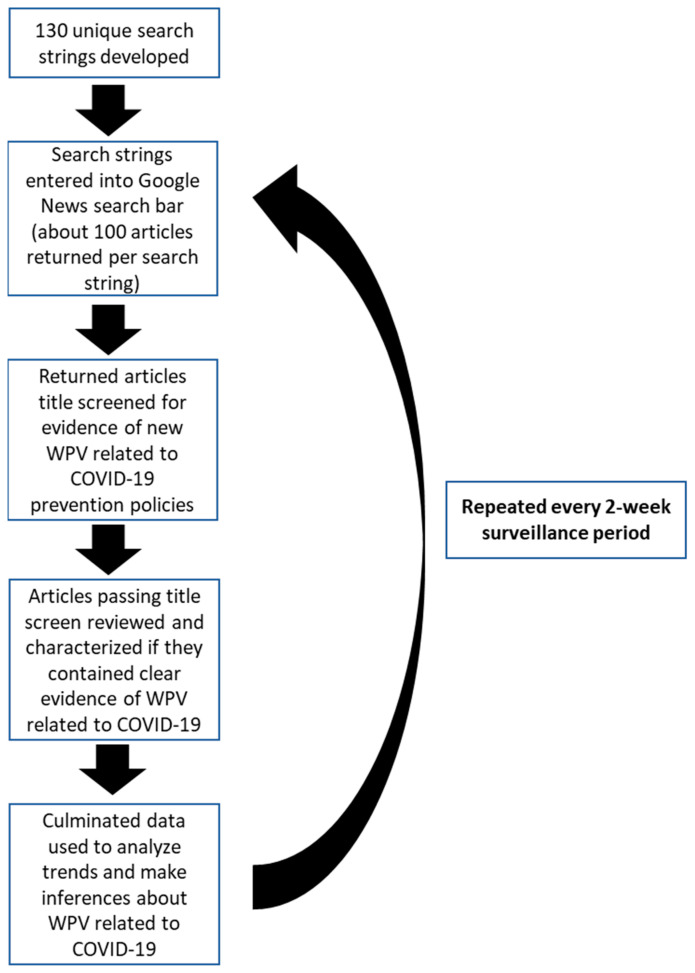
Workplace Violence Related to the COVID-19 Pandemic, United States, News Selection Process.

For this study, a group of experts in WPV and informatics developed a set of 130 unique search queries using combinations of terms specific to COVID-19 (i.e., “Coronavirus” or “COVID-19” or “SARS-CoV-2”), an “action” key term or truncated key term associated with violence (e.g., “assault*”, “harass”, “violen*”, “stab*”), and a subject or location key term associated with potential victims, perpetrators, or items that might cause conflict, including “customer”, “mask”, “worker”, “employee”, “nurse”, “store”. These terms were used by a contractor experienced in conducting reviews using the Google News aggregator service. Google News is the largest news-aggregation service, covering thousands of publishers in multiple languages [13]. Articles obtained included publications by large-scale national media platforms such as CNN, as well as smaller-scale news sources at the regional and local level. Searches were repeated every two weeks. An initial screen of article titles was performed. Articles containing WPV-relevant titles were further reviewed by at least one research assistant who worked for the contractor for any stories suspected of documenting an incident of WPV related to COVID-19 prevention policies or describing an intent to spread (or threat to spread) SARS-CoV-2. Figure 1 provides an overview of the search process.

To be included, the news stories identified in the search had to be published from 1 March 2020, to 31 August 2021, in the U.S. media. We were interested in physical assaults, threats (i.e., verbal, written, and physical expressions that could reasonably be interpreted as intending to cause harm), as well as verbal assaults (i.e., yelling, swearing, insulting, or bullying another person with the intent of hurting or causing harm). We categorized biological violence (e.g., spitting on or at someone, or intentionally coughing at or close to someone’s face) separately from other forms of physical violence for this study. Types of events kept for analysis included customers assaulting or harassing employees; property damage; employees assaulting or harassing other employees; virtual threats if the threat was clearly directed at an employee or business; or workers being assaulted or threatened outside of the workplace if it was related to a professional stigmatization (such as a nurse wearing medical scrubs in public). We excluded events that did not meet our definition such as events where customers assaulted or threatened other customers but there was no clear employee involvement; property was destroyed but there was no threat directed at employees; a violent event was not clearly described; and employees assaulted or threatened a customer and the situation did not involve a direct or indirect threat of another employee.

After each search, all newly identified stories were cross-referenced with previously collected events to ensure events were not duplicated (e.g., when the same event was reported in multiple news outlets). If an article identified a WPV event, relevant variables were abstracted and entered into a Microsoft^®^ Excel^®^ (version 2202, Build 16.0.14931.20764, Microsoft^®^ for Windows^®^, Redmond, U.S.) database. Variables of interest were determined by members of the NIOSH project team and provided to the contractor. Recorded variables for each event included: article title; the Uniform Resource Locator (URL); date of event; location (i.e., city and state); industry where event took place (e.g., retail, food services); perpetrator-victim relationship (e.g., customer-employee, law enforcement-arrestee); instigating factors for violence (e.g., policy enforcement disagreement, criminal intent); age/sex of victim(s) and perpetrator(s); number of victims and perpetrators; and type of violence (e.g., biological assault, verbal harassment, physical assault). Other media sources were searched for missing information if key variables (e.g., date; location; and either name of the victim, name of the perpetrator, or key descriptions of the incident) matched. All information collected by the contractor was reviewed and verified by at least two members of the NIOSH project team. During the NIOSH review, all cases were reviewed to confirm that they met case inclusion criteria. Once cases were confirmed, members of the NIOSH team reviewed existing variables and added variables to identify cases where the dispute was specifically due to a mask mandate and/or a vaccine mandate.

Excel^®^ was used to analyze the data. Excel^®^ pivot tables were used to obtain frequencies and percentages for select variables including details about the victim(s) and the perpetrator(s), instigating factors that led to the event, and the industry in which the event occurred. For each event, the type of violence (physical, non-physical, or biological) was identified. Events may have involved more than one type of event. Thus, the sum of physical, non-physical, and biological acts is likely greater than the total events.

Finally, we also compared events identified for the current study with those identified and described by Tiesman et. al. [12]. The Tiesman et al. study used multiple search engines to improve coverage and reduce bias from the use of a single site. In addition to searching current stories, Tiesman et. al. also used Buzzsumo.com because it is one of the largest and most comprehensive vendors of historical news archives. This retrospective, multi-engine search identified events that occurred from March 2020 to October 2020. Conversely, events for the current study were identified through a biweekly search of a single aggregator service to identify new incidents. We broadly compared results from both studies. We also conducted a more detailed comparison by attempting to match events from the period where the two studies overlapped, March 2020 to October 2020. We compared all the COVID-19 related WPV events for the 8-month period that were identified through both studies. Cases were manually matched, first by state, date, and event details (such as exact business name and location, names of arrested individuals, and descriptions of the incident). Two members of the project team reviewed the matched and unmatched cases and discussed discrepancies to verify the matches. For the detailed match, we determined the percentage of cases that matched, the percentage of cases that should have matched but did not (i.e., they appeared to have overlapping inclusion criteria), and the percentage of cases that did not match because of differing case inclusion criteria.

## 3. Results

### 3.1. Time Trends

**Figure 2:**
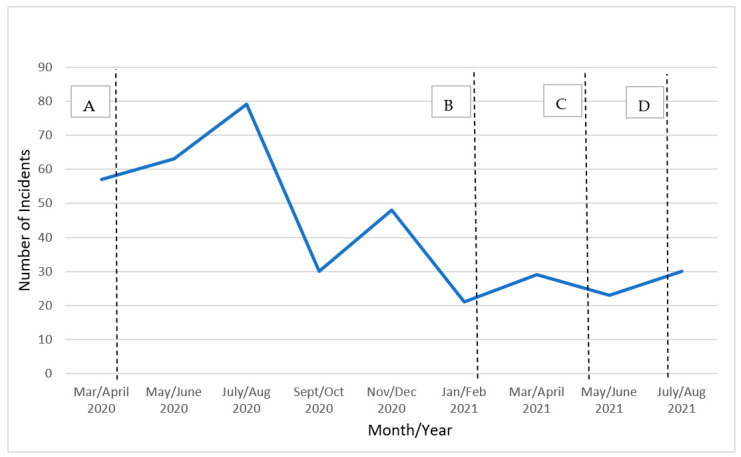
Occurrences of Workplace Violence Related to the COVID-19 Pandemic, United States, Identified in Media from March 2020 to August 2021.

Of the 481 cases initially identified by the contractor, the NIOSH team determined that 408 unique WPV events occurred between 1 March 2020, and 31 August 2021, and met the established case criteria. During this period, there were at least 20 COVID-19 related acts of WPV identified from media reports for each two-month period (Figure 2). The number of WPV events related to COVID-19 peaked in July/August 2020 and then again in November/December 2020.

A. 3 April 2020: CDC recommends universal masking indoors in public or outdoors when social distancing is not possible https://time.com/5794729/coronavirus-face-masks/. (accessed on 1 November 2022).
B. February 2021: CDC recommends double-masking or using mask-fitters indoors in public or outdoors when social distancing is not possible https://www.factcheck.org/2021/03/scicheck-the-evolving-science-of-face-masks-and-covid-19/. (accessed on 1 November 2022).
C. 13 May 2021: CDC Director Dr. Walensky states that fully vaccinated individuals can stop wearing masks indoors https://www.npr.org/2021/05/13/996582891/fully-vaccinated-people-can-stop-wearing-masks-indoors-and-outdoors-cdc-says (accessed on 1 November 2022).
D. 27 July 2021: CDC recommends that all individuals wear masks indoors in areas of substantial or high transmission https://www.usnews.com/news/health-news/articles/2021–07-29/following-cdc-guidance-reversal-will-mask-mandates-make-a-comeback. (accessed on 1 November 2022).

### 3.2. Incident Events: Victims, Perpetrators, and Instigating Factors

Of the 408 WPV events related to the COVID-19 pandemic, workers were victims in most (81%) of the events; both worker(s) and other customer(s)/client(s)/patient(s) were victims in 8%; customers/clients/patients only were victims when workers were present but not targeted in 5%; and only property damage occurred in 6% of events (Table 1). In 55% of the events, a single person experienced a WPV event related to COVID-19; in 35% of the events, multiple persons experienced WPV. Media stories did not always identify the sex of the victim (unidentified in approximately 42% of media reports). Available information indicated that 31% of the victims were identified as males while 18% were identified as females.

Most of the 408 violent events related to COVID-19 involved physical or non-physical violence (Table 1). In WPV events involving physical and non-physical violence, larger proportions of events (64% and 54%, respectively) involved one victim while a larger portion of biological incidents (48%) involved multiple victims. The largest proportions of all three types of violence were experienced by male victims (Table 1). Of the 408 events, 100 events (25%) involved multiple types of violence. Of these 100 events, 53 events involved physical and non-physical violence, 13 involved biological and non-physical violence, 28 events involved biological and physical violence, and 6 events involved all three types of violence (data not shown). The remaining 307 events only referred to one type of violence.

In three quarters of the events, perpetrators were customers/clients/patients while other people from the general public (e.g., citizens attacking public officials during public meetings or at public venues like restaurants) were perpetrators in 13% (Table 2). Most (83%) only involved one perpetrator and males accounted for 60% of the perpetrators. Perpetrators of all three types of violence (i.e., physical, non-physical, and biological) were more often customers/clients/patients (Table 2).

Almost two-thirds (64%) of the events involved a dispute over wearing a mask or face covering (Table 2). Similarly, 71% of the WPV events related to COVID-19 resulted from a policy enforcement disagreement, including disputes over wearing a mask, while 9% of the events resulted from the perpetrator intending to cause fear and/or threaten to spread COVID-19. The largest proportions of physical (78%) and non-physical (78%) violence involved policy enforcement disagreements. Most biological violence events were associated with two instigating factors, policy enforcement disagreements (46%) and the intent of causing fear and/or threatening to spread COVID-19 (25%).

### 3.3. WPV by Industry

By industry, most WPV events occurred in retail or food service (Table 3). Larger proportions of WPV events in retail (56%), food service (53%), transportation (69%), and healthcare (58%) involved one victim whereas a larger proportion (57%) of events in law enforcement involved multiple victims. Larger proportions of victims in retail (24%), food service (31%), law enforcement (21%), and transportation (46%) were male while a larger proportion of victims in healthcare (35%) were female.

Customers/clients/patients were the primary perpetrators in retail (96%), food service (95%), transportation (95%), and healthcare (77%), while arrestees were the primary perpetrators in law enforcement (93%) (Table 4). Male perpetrators accounted for larger proportions in all five industries.

Similarly, the largest percentage of WPV events resulted from policy enforcement disagreements including disputes over masks in retail (78%), food service (88%), and transportation (87%). Over a quarter of the WPV events in healthcare (27%) were instigated with a reported intention of causing fear and/or spreading COVID-19.

### 3.4. Media Scraping Method Comparison

The current study began about the same time as the study conducted by Tiesman et. al. [12] as described in the introduction. The latter study included WPV events occurring between 1 March 2020 and 31 October 2020, while the current study continued through 31 August 2021, as part of a larger literature review effort. A broad comparison of the findings found that both studies saw a peak in WPV cases reported in the media in July 2020, but reasons for this peak are not immediately clear. Also, both studies saw similar perpetrator and victim characteristic profiles. For example, 79% of the perpetrators acted alone in the Tiesman et. al. study and 82% of the perpetrators in the current study also acted alone. Finally, both studies reported similar results by industry. The current study found 19% of the WPV events occurred in food service, 6% in healthcare, 10% in transportation, 11% in law enforcement, and 38% in retail. The Tiesman et. al. study similarly reported 19% of WPV events occurred in food service, 4% in healthcare, 15% in other (most were transportation), 9% in public safety (most were law enforcement), and 48% in retail. Finally, 72% of the WPV events in the Tiesman et. al. study were due to a mask dispute compared to 74% in the current study.

There were some noteworthy differences as well. The Tiesman et. al. study found that in 52% of the events, the only victim was the worker, while the current study reported that the only victim was the worker in 81% of the events. Also, albeit a small number of cases were identified, the current study was able to measure WPV due to vaccination disputes whereas the Tiesman et. al. study could not because vaccines were not available until January of 2021. Finally, the Tiesman et. al. study found that after the July peak, WPV events reported in the media tapered off. The current study suggests that a second smaller peak occurred in November/December 2020, and the numbers further tapered off but ranged from 20 to 30 for each two-month period until August 2021.

Based on a manual one-to-one comparison of data from the two studies from March to October 2020 for which the case definitions matched, a total of 131 events were identified by both media scraping approaches. However, based on a review of the events where the case definition matched, we counted 94 events identified by the current study that were not identified by Tiesman et. al., and 160 events identified by Tiesman et. al., that were not identified by the current study.

## 4. Discussion

### 4.1. Interpreting the Frequency of WPV Related to COVID-19

The number of events identified for this study likely represent only a fraction of events involving WPV related to COVID-19. One study reported findings from 1675 voluntary survey responses of employed applicants to the One Fair Wage Emergency Fund from October 20 to November 10, 2020 [6]. This survey found that 78% of workers in the five states surveyed reported experiencing or witnessing hostile behavior from customers in response to staff enforcing COVID-19 safety protocols; 58% reported feeling reluctant to enforce COVID-19 protocols over concerns of customer retaliation (either hostility, violence, or withholding tips); 59% reported experiencing hostile incidents at least weekly; and 41% reported a noticeable increase in the frequency of unwanted sexual comments from customers during the pandemic [6].

COVID-19-related WPV events increased during the first year of the pandemic. These increases appeared to loosely coincide with the timing of CDC’s recommendations that masks be worn indoors in public places, or outdoors when physical distancing was not possible (Figure 1) [14,15,16,17]. However, we were not able to investigate a causal relationship. Furthermore, the lower numbers of WPV events from January/February 2021 through July/August 2021 coincide with the introduction of vaccinations, as well as CDC’s recommendation that fully vaccinated individuals could stop wearing masks indoors (Figure 1); however, it is unclear whether these recommendations had direct influence on the number of monthly events since data on the prevalence of workplace-specific masking policies or whether staff routinely enforced policies were not available.

The decline in numbers of WPV events reported in the media later in the pandemic might reflect a true decline in events as people became more accustomed to COVID-19 prevention policies and masking—or it might reflect that (1) events declined as workplaces stopped trying to enforce mask mandates to prevent altercations, or (2) events did not decline, but media interest in covering such events decreased.

### 4.2. COVID-19 Related WPV by Sex

A pre-pandemic analysis of WPV events from the National Crime Victimization Survey (NCVS) for data years 2007–2015 reported that 70% of the perpetrators of violence were males, while females accounted for 14%, and events with both male and female perpetrators accounted for 4% of the incidents [18]. Our media-based evaluation varied somewhat with 60% of the perpetrators being male, and the percentage of females (27%) almost double that of the earlier NCVS data, while events with both male and female perpetrators accounted for 3%. Almost two-thirds (63%) of the victims in the pre-pandemic NCVS study were males, while 37% were females. Our media-based study reported a large proportion (42%) of events where the gender of the victims was unknown, with only 31% of the victims identified as male and 18% identified as females.

### 4.3. COVID-19 Related WPV by Industry

Very few of the media reports involved healthcare; this might reflect that such conflicts were not unusual or are not sensational enough to attract media attention. For at least a decade before the COVID-19 pandemic began, injuries related to violence increased among healthcare workers in the U.S. [19]. This trend appears to be continuing in both the U.S. and abroad; the International Committee of the Red Cross (ICRC) raised alarms in August of 2020 that violence, harassment, and stigmatization against healthcare workers appeared to be increasing worldwide [20]. Several recent studies have shown that violence and harassment against healthcare professionals have been frequent during the COVID-19 pandemic [21,22,23,24,25,26]. While one of these studies was based on data collected in the U.S., most of these studies come from low to middle-income countries.

Data from the Bureau of Labor Statistics’ Survey of Occupational Injuries and Illnesses (SOII) over the last decade has consistently reported 70% or more of the WPV related injuries requiring days away from work to recover are sustained by workers in the healthcare and social assistance (HCSA) industry. In 2019, 70% (*n* = 14,550 of 20,870) of the total injuries reported in the SOII from WPV occurred in the HCSA industry [27]. In 2020, this increased to 76% (*n* = 15,210 of 20,050) of WPV [28]. Although an in-depth analysis of this database will be necessary to determine if the increase can be attributed to COVID-19 related violence, the discrepancy between WPV in the SOII compared to media scrapings suggests that violence in healthcare settings is occurring but less likely to be reported in the media.

The pre-pandemic analysis of NCVS data from 2007–2015 showed that non-fatal WPV affecting workers was most common among protective services workers (101.4 events per 1000 workers), followed by community and social assistance workers (19.1 per 1000), healthcare practitioners (17.1 per 1000) and healthcare support workers (16.5 per 1000) [18]. Crime victimization was far less common among food preparation and serving workers (at 5.8 events per 1000 workers) and sales and related workers (5.3 per 1000 workers) [18]. Our evaluation of media reports of WPV events involving COVID-19 flips these pre-pandemic trends, finding more media reports of WPV among retail or food service and fewer media reports of WPV among healthcare personnel.

### 4.4. Historical Precedents for Violence during Epidemics

The finding that violence occurred against workers related to the pandemic and efforts to control the spread of the virus should not be surprising. Epidemics of the recent and more distant past have involved similar events. Cohn and Kutalek [4] described a wide range of violence directed at public health and healthcare workers during the 2014 Ebola virus disease (EVD) outbreak in Africa, including incidents such as rocks thrown at Red Cross vehicles, attacks on staff at a newly constructed EVD clinic, threats against local officials, and even the murder of eight members of a delegation of physicians, politicians, and journalists. Historical records revealing mistrust, hostility and overt violence against workers involved in infection control or other public health activity can be found as far back as the Cholera wave of the 1830s in Europe and during each of the subsequent waves up to the last outbreak in 1911 [4]. These epidemics, the records note, fostered widespread hostility toward the health care and public health communities, and these sentiments were often interlinked with perceptions that inequitable conditions of societies had greater impact on the lower socioeconomic classes [4].

Likewise, there are parallels to the rise in violence during the COVID-19 pandemic and the 1918 influenza pandemic. Dolan [5] describes how efforts to mandate wide scale masking requirements led to both organized opposition as well as episodic violence against those attempting to enforce these public health mandates. For example, tensions around mask mandates in San Francisco (which passed a mask mandate in 1918) led to hundreds of arrests in the city, with events including conflicts between pro- and anti-maskers, as well as run-ins between citizens and police and public health workers. The history of infectious disease epidemics from cholera and the 1918 influenza pandemic through Ebola and COVID-19 reveal an increase in societal tensions and violence during each of these pandemics, with workers involved in policy enforcement, infection control, and health care often bearing the brunt.

## 5. Study Limitations

The data from this study provided important information on WPV events related to COVID-19 and gave us the opportunity to rapidly explore characteristics of these events to gain a better understanding of why they were occurring. However, there are a few limitations that should be considered. First, results presented in this study should be considered an underestimate of the actual number of events that occurred from March 2020 through August 2021. Furthermore, the findings in this study may not be generalizable beyond the events that were identified. The underestimation and lack of generalizability are largely due to the inherent biases and limitations of the source of the events. Cases included in this study were from media reports and not from a comprehensive survey or reporting system. While we created a consistent approach to identifying events, the mix of physical versus non-physical WPV events related to COVID-19 captured for this study likely followed the media’s tendency to report events that garner the most attention. Under normal circumstances, acts of physical violence are usually the most frequently reported incidents as they attract the most readers and allow the news media to make a statement about violence. Furthermore, in the case of non-physical violence and minor physical violence, these types of events are often handled internally and not reported to the police or the media. Secondly, while this study found a decrease in the number of events during the study period, the trend may reflect the media tendency and interest to report these events. We were not able to assess whether the trend was a true decrease or simply that the novelty of these events wore off as the pandemic continued.

A final limitation is inherent to the use of media scrapings for such events. On one hand, the Tiesman et al., and the current studies suggest that using such a tool for something other than disease detection is generally feasible. While the broad comparison of findings from the two studies identified many similarities, the manual one-to-one match suggested that there may have been issues with identifying events that could not be fully understood. The Tiesman et. al., study did not appear to lead to increased completeness of surveillance, given that this tactic only found 57% of the incidents identified by the rapid method described in the current study. A much more extensive comparison would need to be conducted to assess the exact reasons, but this may be due, in part, to media sites taking down content over time. We noted in our review that older content, especially for small or local media stations, had been removed from its original posted URL and could only be obtained from pay-to-view online archives. The difference may also be due to the prioritization of results from search engines changing over time, with some reports dropping further down or possibly out of the results entirely. If similar studies are conducted, more emphasis should be placed on understanding how events are identified through search engines and options to cost effectively optimize findings.

## 6. Conclusions

Prior to the pandemic, progress had been made in preventing WPV [9]. As the COVID-19 pandemic continued, issues surrounding WPV were likely intensified as essential workers faced rapid changes in work environments as they adapted to and often had to enforce new infection prevention and control policies [29]. During times of crises like the COVID-19 pandemic, systematically collected sources of data may not be readily available. This study and the Tiesman et al. [12] study have both demonstrated that media scrapings to identify workplace events such as WPV may be a useful approach when other sources of data are not readily available. 

To understand WPV related to COVID-19 prevention policies, the data collected through this study provided NIOSH with a near-real time glimpse into WPV events related to COVID-19 prevention policies and timely identification of high risk worker groups. These data suggest that WPV related to COVID-19 prevention policies were more frequent among retail or food service and that WPV was more frequently perpetrated by customers/clients/patients. Furthermore, WPV events occurred most often when workers were enforcing workplace or government COVID-19 policies. The results suggest that the distribution of WPV events among industries during pandemics and other times of crises may differ from non-pandemic times. As employers seek to improve workplace safety and ensure that WPV prevention programs are adaptable, these data may provide a basis for improvements in WPV prevention and intervention. Further research is needed to understand if existing prevention resources such as training and education [7,30,31] were offered as staff were asked to enforce company, local, or state requirements.

## Figures and Tables

**Table 1:** Victim Characteristics and Type of Violence among Workplace Violence Events Related to the COVID-19 Pandemic in United States, Identified in Media from March 2020 to August 2021.

	Total Violent Events	Physical Violence *	Non-Physical Violence *	Biological *^Ɨ^
	*N* (%)	*N* (%)	*N* (%)	*N* (%)
**Total**	408 (100%) ^ǂ^	195 ^ǂ^	192 ^ǂ^	129 ^ǂ^
**Victim of Event**				
Worker	331 (81%)	157 (81%)	158 (82%)	104 (81%)
Customer/Client/Patient	19 (5%)	12 (6%)	7 (4%)	4 (3%)
Both worker and customer/client/patient	32 (8%)	9 (5%)	19 (10%)	15 (12%)
Not applicable (property damage)	24 (6%)	15 (8%)	6 (3%)	6 (5%)
Unknown	2 (<1%)	2 (1%)	2 (1%)	0 (0%)
**Number of Victims**				
Single	225 (55%)	124 (64%)	103 (54%)	55 (43%)
Multiple	142 (35%)	53 (27%)	70 (36%)	62 (48%)
Not applicable (property damage)	24 (6%)	15 (8%)	6 (3%)	6 (5%)
Unknown	17 (4%)	3 (2%)	13 (7%)	6 (5%)
**Gender of Victims**				
Male	125 (31%)	74 (38%)	63 (33%)	25 (19%)
Female	74 (18%)	35 (18%)	36 (19%)	22 (17%)
Male and Female (multiple victims)	12 (3%)	5 (3%)	8 (4%)	3 (2%)
Not applicable (property damage)	24 (6%)	15 (8%)	6 (3%)	6 (5%)
Unknown	173 (42%)	66 (34%)	79 (41%)	73 (57%)

* For this study, physical violence includes hitting, slapping, pushing, grabbing, or any other action that leads to physical contact with the intention of injuring or causing harm. Non-physical violence includes using words, gestures, yelling, swearing, or other actions with the intent of intimidating or frightening someone [8,12]. ^Ɨ^ Biological violence is coughing, spitting, or some other action that is done to try to infect someone. While biological violence is a form of physical violence, for the purposes of this study, acts of biological violence were categorized separately. ^ǂ^ The sum of the total number of physical, non-physical, and biological acts is greater than the total events as events may have involved more than one type of violence.

**Table 2:** Perpetrator Characteristics and Type of Violence among Workplace Violence Events Related to the COVID-19 Pandemic in United States, Identified in Media from March 2020 to August 2021.

	Total Violent Events	Physical Violence *	Non-Physical Violence *	Biological *^Ɨ^
	*N* (%)	*N* (%)	*N* (%)	*N* (%)
**Total**	408 (100%) ^ǂ^	195 ^ǂ^	192 ^ǂ^	129 ^ǂ^
**Perpetrator of Event** **^§^**				
Customer/Client/Patient	305 (75%)	165 (85%)	137 (71%)	90 (70%)
General Public	51 (13%)	17 (9%)	39 (20%)	4 (3%)
Arrestee	39 (10%)	9 (5%)	9 (5%)	31 (24%)
Worker	12 (3%)	3 (2%)	6 (3%)	4 (3%)
**Number of Perpetrators**				
Single	338 (83%)	162 (83%)	147 (77%)	114 (88%)
Multiple	66 (16%)	32 (16%)	42 (22%)	14 (11%)
Unknown	4 (1%)	1 (<1%)	3 (2%)	1 (1%)
**Gender of Perpetrators**				
Male	244 (60%)	125 (64%)	112 (58%)	71 (55%)
Female	110 (27%)	49 (25%)	43 (22%)	48 (37%)
Male and Female (multiple perps)	14 (3%)	7 (4%)	9 (5%)	6 (5%)
Unknown	40 (10%)	14 (7%)	28 (15%)	4 (3%)
**Did the incident involve a mask dispute?**				
Yes	262 (64%)	143 (73%)	142 (74%)	46 (36%)
**Did the incident involve a vaccination dispute?**				
Yes	10 (2%)	3 (2%)	7 (4%)	0 (0%)
**Instigating Factors** **^||^**				
Policy Enforcement Disagreement	288 (71%)	153 (78%)	149 (78%)	59 (46%)
Intent of causing fear and/or spreading (or threatening to spread) COVID	35 (9%)	3 (2%)	5 (3%)	32 (25%)
Resisting Arrest	16 (4%)	8 (4%)	3 (2%)	14 (11%)
Mask or Professional Stigmatization	15 (4%)	8 (4%)	9 (5%)	1 (<1%)
COVID-Related Stress including fear of getting COVID	13 (3%)	6 (3%)	6 (3%)	3 (2%)
Xenophobically Motivated	11 (3%)	6 (3%)	9 (5%)	1 (<1%)
Criminal Intent	8 (2%)	4 (2%)	4 (2%)	3 (2%)
General Dispute	6 (1%)	1 (<1%)	3 (2%)	4 (3%)

* For this study, physical violence includes hitting, slapping, pushing, grabbing, or any other action that leads to physical contact with the intention of injuring or causing harm. Non-physical violence includes using words, gestures, yelling, swearing, or other actions with the intent of intimidating or frightening someone [8,12] ^Ɨ^ Biological violence is coughing, spitting, or some other action that is done to try to infect someone. While biological violence is a form of physical violence, for the purposes of this study, acts of biological violence were categorized separately. ^ǂ^ The sum of the total number of physical, non-physical, and biological acts does not add up to the total number of events as events may have involved more than one type of violence. Thus, the sum of physical, non-physical, and biological acts is greater than the total events. ^§^ One event did not have enough detail to determine who the perpetrator was. ^||^ There were 16 events where the instigating factor was not known. Thus, the column values will not add to the column total.

**Table 3:** Victim Characteristics by Select Industries * among Workplace Violence Events Related to the COVID-19 Pandemic in United States, Identified in Media from March 2020 to August 2021.

	Retail	Food Service	Law Enforcement	Transportation	Healthcare
	*N* (%)	*N* (%)	*N* (%)	*N* (%)	*N* (%)
**Total**	156	78	42	39	26
**Victim of Event ^Ɨ^**					
Worker	112 (72%)	63 (81%)	41 (98%)	31 (79%)	26 (100%)
Customer/Client/Patient	12 (8%)	2 (3%)	0 (0%)	3 (8%)	0 (0%)
Both worker and customer/client/patient	18 (12%)	5 (6%)	1 (2%)	3 (8%)	0 (0%)
Not applicable (property damage)	13 (8%)	7 (9%)	0 (0%)	2 (5%)	0 (0%)
**Number of Victims**					
Single	87 (56%)	41 (53%)	13 (31%)	27 (69%)	15 (58%)
Multiple	50 (32%)	26 (33%)	24 (57%)	9 (23%)	11 (42%)
Not applicable (property damage)	13 (8%)	7 (9%)	0 (0%)	2 (5%)	0 (0%)
Unknown	6 (4%)	4 (5%)	5 (12%)	1 (3%)	0 (0%)
**Gender of Victims**					
Male	38 (24%)	24 (31%)	9 (21%)	18 (46%)	3 (12%)
Female	30 (19%)	15 (19%)	2 (5%)	10 (26%)	9 (35%)
Male and Female (multiple victims)	5 (3%)	4 (5%)	0 (0%)	1 (3%)	0 (0%)
Not applicable (property damage)	13 (8%)	7 (9%)	0 (0%)	2 (5%)	0 (0%)
Unknown	70 (45%)	28 (36%)	31 (74%)	8 (21%)	14 (54%)

* Industries not reported here include government (25 events); other services (37 events); public health (3 events); and other/unknown (2 events). ^Ɨ^ One event did not have enough detail to determine who the victim was.

**Table 4:** Perpetrator Characteristics by Select Industries * among Workplace Violence Events Related to the COVID-19 Pandemic in United States, Identified in Media from March 2020 to August 2021.

	Retail	Food Service	Law Enforcement	Transportation	Healthcare
	*N* (%)	*N* (%)	*N* (%)	*N* (%)	*N* (%)
**Total**	156	78	42	39	26
**Perpetrator of Event ^Ɨ^**					
Customer/Client/Patient	150 (96%)	74 (95%)	2 (5%)	37 (95%)	20 (77%)
General Public	5 (3%)	2 (3%)	0 (0%)	1 (3%)	5 (19%)
Arrestee	0 (0%)	0 (0%)	39 (93%)	0%	0 (0%)
Worker	1 (<1%)	1 (1%)	1 (2%)	1 (3%)	1 (4%)
**Number of Perpetrators ^ǂ^**					
Single	131 (84%)	61 (78%)	41 (98%)	33 (85%)	24 (92%)
Multiple	25 (16%)	13 (17%)	1 (2%)	6 (15%)	2 (8%)
**Gender of Perpetrators**					
Male	100 (64%)	40 (51%)	25 (60%)	23 (59%)	15 (58%)
Female	38 (24%)	23 (29%)	16 (38%)	14 (36%)	9 (35%)
Male and Female (multiple perps)	8 (5%)	4 (5%)	0 (0%)	1 (3%)	0 (0%)
Unknown	10 (6%)	11 (14%)	1 (2%)	1 (3%)	2 (8%)
**Did the incident involve a mask dispute?**					
Yes	109 (70%)	59 (76%)	10 (24%)	33 (85%)	8 (31%)
**Instigating Factors** **^§^**					
Policy Enforcement Disagreement	122 (78%)	69 (88%)	8 (19%)	34 (87%)	9 (35%)
Intent of causing fear and/or spreading (or threatening to spread) COVID	10 (6%)	0 (0%)	17 (40%)	0 (0%)	7 (27%)
Resisting Arrest	0 (0%)	0 (0%)	15 (36%)	0 (0%)	1 (4%)
Mask or Professional Stigmatization	3 (2%)	0 (0%)	0 (0%)	0 (0%)	3 (12%)
COVID-Related Stress including fear of getting COVID	3 (2%)	1 (1%)	1 (2%)	1 (3%)	1 (4%)
Criminal Intent	5 (3%)	1 (1%)	1 (2%)	0 (0%)	1 (4%)
Xenophobically Motivated	2 (1%)	4 (5%)	0 (0%)	1 (3%)	1 (4%)
General Dispute	2 (1%)	1 (1%)	0 (0%)	1 (3%)	1 (4%)

* Industries not reported here include government (25 events); other services (37 events); public health (3 events); and other/unknown (2 events). ^Ɨ^ For the food service industry, one event did not have enough detail to determine who the perpetrator was. ^ǂ^ For the food service industry, four events did not have enough detail to determine the number of perpetrators involved. **^§^** There were 16 events where the instigating factor was not known. Thus, the column values will not add to the column total.

## Data Availability

These data were derived from public domain news services identified via the Google News Aggregator. Derived data supporting the findings of this study are available from the corresponding author on reasonable request.
